# Supplementary material for: Rab12 is a regulator of mitophagy and mitochondrial homeostasis
Source: bioRxiv. 2026 Mar 31:2026.03.29.715103. Preprint. [Version 1] doi: 10.64898/2026.03.29.715103 (PMC13060255; doi:10.64898/2026.03.29.715103)
Supplement: Supplement 1 [file NIHPP2026.03.29.715103v1-supplement-1.pdf]

bioRxiv preprint doi: https://doi.org/10.64898/2026.03.29.715103; this version posted March 31, 2026. The copyright holder for this preprint (which was not certified by peer review) is the author/funder, who has granted bioRxiv a license to display the preprint in perpetuity. It is made available under aCC-BY 4.0 International license.

SUPPLEMENTARY MATERIAL

bioRxiv preprint doi: https://doi.org/10.64898/2026.03.29.715103; this version posted March 31, 2026. The copyright holder for this preprint (which was not certified by peer review) is the author/funder, who has granted bioRxiv a license to display the preprint in perpetuity. It is made available under aCC-BY 4.0 International license.

Supplementary Table 1: Endogenous mRNA expression levels of Rab family protein in HeLa. Gene RNA expression levels, presented as normalized transcripts per million (nPTM), of each Rab GTPase family member analyzed through siRNA screening according to the Human Protein Atlas.[25]

Gene Name	Expression levels in HeLa (nPTM)	Protein Atlas URL
Rab1A	135.5	https://www.proteinatlas.org/ENSG00000138069-RAB1A
Rab1B	190.9	https://www.proteinatlas.org/ENSG00000174903-RAB1B
Rab2A	65.7	https://www.proteinatlas.org/ENSG00000104388-RAB2A
Rab2B	6.1	https://www.proteinatlas.org/ENSG00000129472-RAB2B
Rab3A	6.3	https://www.proteinatlas.org/ENSG00000105649-RAB3A
Rab3B	1.1	https://www.proteinatlas.org/ENSG00000169213-RAB3B
Rab3C	1.7	https://www.proteinatlas.org/ENSG00000152932-RAB3C
Rab3D	6.6	https://www.proteinatlas.org/ENSG00000105514-RAB3D
Rab4A	11.1	https://www.proteinatlas.org/ENSG00000168118-RAB4A
Rab4B	6.0	https://www.proteinatlas.org/ENSG00000167578-RAB4B
Rab5A	31.5	https://www.proteinatlas.org/ENSG00000144566-RAB5A
Rab5B	44.5	https://www.proteinatlas.org/ENSG00000111540-RAB5B
Rab5C	165.4	https://www.proteinatlas.org/ENSG00000108774-RAB5C
Rab6A	69.4	https://www.proteinatlas.org/ENSG00000175582-RAB6A
Rab6B	2.4	https://www.proteinatlas.org/ENSG00000154917-RAB6B
Rab6C	Not detected	https://www.proteinatlas.org/ENSG00000222014-RAB6C
Rab7A	100.9	https://www.proteinatlas.org/ENSG00000075785-RAB7A
Rab7B	Not detected	https://www.proteinatlas.org/ENSG00000276600-RAB7B
Rab8A	73.4	https://www.proteinatlas.org/ENSG00000167461-RAB8A
Rab8B	7.7	https://www.proteinatlas.org/ENSG00000166128-RAB8B
Rab9A	34.4	https://www.proteinatlas.org/ENSG00000123595-RAB9A
Rab9B	Not detected	https://www.proteinatlas.org/ENSG00000123570-RAB9B
Rab10	58.6	https://www.proteinatlas.org/ENSG00000084733-RAB10
Rab11A	277.9	https://www.proteinatlas.org/ENSG00000103769-RAB11A
Rab11B	88.2	https://www.proteinatlas.org/ENSG00000185236-RAB11B
Rab12	22.1	https://www.proteinatlas.org/ENSG00000206418-RAB12
Rab13	76.6	https://www.proteinatlas.org/ENSG00000143545-RAB13

bioRxiv preprint doi: https://doi.org/10.64898/2026.03.29.715103; this version posted March 31, 2026. The copyright holder for this preprint (which was not certified by peer review) is the author/funder, who has granted bioRxiv a license to display the preprint in perpetuity. It is made available under aCC-BY 4.0 International license.

Rab14	95.8	https://www.proteinatlas.org/ENSG00000119396-RAB14
Rab15	1.2	https://www.proteinatlas.org/ENSG00000139998-RAB15
Rab17	0.2	https://www.proteinatlas.org/ENSG00000124839-RAB17
Rab18	66.7	https://www.proteinatlas.org/ENSG00000099246-RAB18
Rab19	0.3	https://www.proteinatlas.org/ENSG00000146955-RAB19
Rab20	4.8	https://www.proteinatlas.org/ENSG00000139832-RAB20
Rab21	3.7	https://www.proteinatlas.org/ENSG00000080371-RAB21
Rab22A	10.4	https://www.proteinatlas.org/ENSG00000124209-RAB22A
Rab23	11.6	https://www.proteinatlas.org/ENSG00000112210-RAB23
Rab24	20.3	https://www.proteinatlas.org/ENSG00000169228-RAB24
Rab25	Not detected	https://www.proteinatlas.org/ENSG00000132698-RAB25
Rab26	12.3	https://www.proteinatlas.org/ENSG00000167964-RAB26
Rab27A	30.4	https://www.proteinatlas.org/ENSG00000069974-RAB27A
Rab27B	3.5	https://www.proteinatlas.org/ENSG00000041353-RAB27B
Rab28	19.1	https://www.proteinatlas.org/ENSG00000157869-RAB28
Rab7L1 (Rab29)	10.3	https://www.proteinatlas.org/ENSG00000117280-RAB29
Rab30	16.9	https://www.proteinatlas.org/ENSG00000137502-RAB30
Rab31	116.8	https://www.proteinatlas.org/ENSG00000168461-RAB31
Rab32	132.3	https://www.proteinatlas.org/ENSG00000118508-RAB32
Rab33A	0.3	https://www.proteinatlas.org/ENSG00000134594-RAB33A
Rab33B	4.1	https://www.proteinatlas.org/ENSG00000172007-RAB33B
Rab34	154.4	https://www.proteinatlas.org/ENSG00000109113-RAB34
Rab35	36.1	https://www.proteinatlas.org/ENSG00000111737-RAB35
Rab36	0.3	https://www.proteinatlas.org/ENSG00000100228-RAB36
Rab37	0.1	https://www.proteinatlas.org/ENSG00000172794-RAB37
Rab38	5.3	https://www.proteinatlas.org/ENSG00000123892-RAB38
Rab39	Not detected	https://www.proteinatlas.org/ENSG00000179331-RAB39A
Rab39B	Not detected	https://www.proteinatlas.org/ENSG00000155961-RAB39B
Rab40A	1.7	https://www.proteinatlas.org/ENSG00000172476-RAB40A
Rab40B	10.9	https://www.proteinatlas.org/ENSG00000141542-RAB40B
Rab40C	12.3	https://www.proteinatlas.org/ENSG00000197562-RAB40C
Rab41	0.2	https://www.proteinatlas.org/ENSG00000147127-RAB41

bioRxiv preprint doi: https://doi.org/10.64898/2026.03.29.715103; this version posted March 31, 2026. The copyright holder for this preprint (which was not certified by peer review) is the author/funder, who has granted bioRxiv a license to display the preprint in perpetuity. It is made available under aCC-BY 4.0 International license.

Rab42	7.6	https://www.proteinatlas.org/ENSG00000188060-RAB42
Rab43	2.6	https://www.proteinatlas.org/ENSG00000172780-RAB43

bioRxiv preprint doi: https://doi.org/10.64898/2026.03.29.715103; this version posted March 31, 2026. The copyright holder for this preprint (which was not certified by peer review) is the author/funder, who has granted bioRxiv a license to display the preprint in perpetuity. It is made available under aCC-BY 4.0 International license.

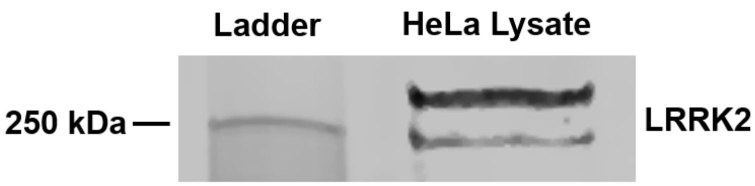

Supplementary Figure 1: LRRK2 protein is expressed in mt-mKeima/YFP-Parkin HeLa cells. Western immunoblot of untreated mt-mKeima/YFP-Parkin HeLa cell lysates was probed with rabbit anti-LRRK2 to show endogenous LRRK2 expression.

bioRxiv preprint doi: https://doi.org/10.64898/2026.03.29.715103; this version posted March 31, 2026. The copyright holder for this preprint (which was not certified by peer review) is the author/funder, who has granted bioRxiv a license to display the preprint in perpetuity. It is made available under aCC-BY 4.0 International license.

Supplementary Table 2: Sequence and catalogue information of Rab family siRNA library. Sequences, Qiagen catalog numbers, and gene identifier information for the siRNA library against human Rab family proteins utilized in mitophagy screening.

Target Sequence	Cat. No.	Product Name	Entrez Gene Id	Gene Symbol
CAGCATGAATCCCGAATATGA	SI00060375	Hs_RAB1A_3	5861	RAB1A
CTGCACTACAACAGATTCTTA	SI00060382	Hs_RAB1A_4		
CTGCAGGAAATAGATCGTTAT	SI02662163	Hs_RAB1A_8		
AACTATAGAGTTAGACGGGAA	SI02662716	Hs_RAB1A_9		
TGCCCTCCAGCTTGTATTTAA	SI00697291	Hs_RAB1B_1	81876	RAB1B
GAGGCTGGGCACCAGCCTTAA	SI00697305	Hs_RAB1B_3		
ACAGCTGCTTCCTGCAAGAAA	SI00697312	Hs_RAB1B_4		
CTGCGGTGGGATCTGAGTATA	SI02662170	Hs_RAB1B_6		
ACGATATTACACGGAGAGATA	SI00044765	Hs_RAB2_2	5862	RAB2A
AGCAGGAGCTTTACTAGTTTA	SI00044779	Hs_RAB2_4		
GGCGACACAGGTGTTGGTAAA	SI00301567	Hs_RAB2_5		
CATGCTTATTGCTACAGTTTA	SI02655023	Hs_RAB2_6		
CTGCTAGTAAGCAGTCACATA	SI00144732	Hs_RAB2B_2	84932	RAB2B
CCCACTAAGAATTAGGGTCAA	SI00144739	Hs_RAB2B_3		
AGGGTTCTTCTTATCCTCCTA	SI02779217	Hs_RAB2B_6		
CAGCGGAACTCTCGTGACATA	SI02779224	Hs_RAB2B_7		
CCCGTCACCCTTATTTATTAT	SI00044786	Hs_RAB3A_1	5864	RAB3A
CCGGACCATCACCACCGCATA	SI00044793	Hs_RAB3A_2		
CAAGATTCTCATCATCGGCAA	SI00044800	Hs_RAB3A_3		
CACCAACGAGGAATCCTTCAA	SI00044807	Hs_RAB3A_4		

bioRxiv preprint doi: https://doi.org/10.64898/2026.03.29.715103; this version posted March 31, 2026. The copyright holder for this preprint (which was not certified by peer review) is the author/funder, who has granted bioRxiv a license to display the preprint in perpetuity. It is made available under aCC-BY 4.0 International license.

CAGCACTAGACTAACATAACA	SI00044814	Hs_RAB3B_1	5865	RAB3B
CGGGTGCAGAATCACTTTACA	SI00044821	Hs_RAB3B_2		
CCGGACCATCACAACAGCCTA	SI00044828	Hs_RAB3B_3		
CAGAGAGCTCAGACTCTCTAA	SI00044835	Hs_RAB3B_4		
GTGGCAAATATGTGATCTTAA	SI00150892	Hs_RAB3C_3	115827	RAB3C
AAGGACATTTGCCAACAATGA	SI00150899	Hs_RAB3C_4		
TACAAGATTGGTCAACTCAAA	SI02779357	Hs_RAB3C_5		
GTGGATATCATCTGCGACAAA	SI03106943	Hs_RAB3C_6		
CAGGCCCTGTTTAGCTGTTTA	SI00062181	Hs_RAB3D_1	9545	RAB3D
CTGGAACTATGGACCACATTA	SI00062195	Hs_RAB3D_3		
AAGCAAGTTCTTGCCCAATCA	SI00062202	Hs_RAB3D_4		
CAAACTGCTACTGATAGGCAA	SI03052595	Hs_RAB3D_6		
CTGATGTATGATATGATAGAA	SI00065471	Hs_RAB4A_2	5867	RAB4A
GACGGCCATGTCCGAAACCTA	SI02655030	Hs_RAB4A_6		
CACCGTTAGATGTGTATGTAA	SI02662226	Hs_RAB4A_7		
AACCTACAATGCGCTTACTAA	SI02662786	Hs_RAB4A_8		
CCGCACTATCCTCAACAAGAT	SI02662793	Hs_RAB4B_6	53916	RAB4B
AAGATTGACTCAGGCGAGCTA	SI03032547	Hs_RAB4B_7		
GACCTACGACTTCCTCTTCAA	SI03101721	Hs_RAB4B_8		
TGGGAAGACTGTGAAGCTACA	SI03120670	Hs_RAB4B_9		
ATCAGTGTTGTAGTAACTAAA	SI02632602	Hs_RAB5A_6	5868	RAB5A
CAAGGCCGACCTAGCAAATAA	SI02632609	Hs_RAB5A_7		
ATAGTTGTATATGATATCACA	SI03046673	Hs_RAB5A_9		

bioRxiv preprint doi: https://doi.org/10.64898/2026.03.29.715103; this version posted March 31, 2026. The copyright holder for this preprint (which was not certified by peer review) is the author/funder, who has granted bioRxiv a license to display the preprint in perpetuity. It is made available under aCC-BY 4.0 International license.

TAGCCTAGCACCAATGTACTA	SI03111115	Hs_RAB5A_10		
CACACTCAATTTGATATCCAT	SI00044849	Hs_RAB5B_2	5869	RAB5B
TTGGTCCTTTCTAAATCTCTA	SI00044863	Hs_RAB5B_4		
CAGAATGCGACTGCTGATTTA	SI02662233	Hs_RAB5B_6		
TAGGTACAAGACAGCGACTTA	SI02662800	Hs_RAB5B_7		
CAGCCGATCGATCGACCTCAA	SI00065583	Hs_RAB5C_2	5878	RAB5C
CACCATGATTTCTCCATATAA	SI02663073	Hs_RAB5C_5		
CCCGACTGGAATCCACTCTAA	SI02663080	Hs_RAB5C_6		
CTGCAATGAACGTGAACGAAA	SI03094161	Hs_RAB5C_7		
ATGGGTGTGATTGTCTTCCTA	SI00044891	Hs_RAB6A_4	5870	RAB6A
CCCACTTATTGTCACCTTGTA	SI02654036	Hs_RAB6A_6		
TACGGTCTTCTTTGAGGTCAA	SI02654120	Hs_RAB6A_7		
CAGATTCATGTATGACAGTTT	SI02655044	Hs_RAB6A_8		
CGGCTGTTACTTAAACAACTA	SI00116963	Hs_RAB6B_1	51560	RAB6B
ATCCATGTTCTTAGAGCCTCA	SI00116970	Hs_RAB6B_2		
ATGGCCAGAGTGGGTCGTCAA	SI00116977	Hs_RAB6B_3		
CAGGGATCACATCACTCTTAA	SI02777705	Hs_RAB6B_6		
AGCACTGATGTAAATTACTTA	SI00142247	Hs_RAB6C_3	84084	RAB6C
TTACTTCTTCATGGTAAGATA	SI03021284	Hs_RAB6C_5		
TTCGAGTTACAGGTCAGGAAA	SI03022754	Hs_RAB6C_6		
TAGCAGAAATAACGATATCTA	SI03110947	Hs_RAB6C_7		
TCCCGTTAGATCAGCATTCTA	SI00066395	Hs_RAB7_2	7879	RAB7A
CTGCTGCGTTCTGGTATTTGA	SI03096016	Hs_RAB7_6		

bioRxiv preprint doi: https://doi.org/10.64898/2026.03.29.715103; this version posted March 31, 2026. The copyright holder for this preprint (which was not certified by peer review) is the author/funder, who has granted bioRxiv a license to display the preprint in perpetuity. It is made available under aCC-BY 4.0 International license.

CACGTAGGCCTTCAACACAAT	SI02662240	Hs_RAB7_5		
GAGGTGGAGCTGTACAACGAA	SI03104255	Hs_RAB7_7		
AACGAGCAGACTAAGGAGTAA	SI00160041	Hs_RAB7B_1	338382	RAB7B
CAGCATCCTCTCCAAGATTAT	SI00160048	Hs_RAB7B_2		
TCGAGGTACCAGAGCATCTTA	SI00160055	Hs_RAB7B_3		
AAGGTGGACCTGAAACTCATT	SI03036502	Hs_RAB7B_5		
CACGTTGTATATTCAGAGAGA	SI00076090	Hs_RAB8A_1	4218	RAB8A
ATGCTCTGTTTACATCGATAA	SI00076104	Hs_RAB8A_3		
TCGCCAGAGATATCAAAGCAA	SI02662254	Hs_RAB8A_5		
CAACGAGAAGTCCTTCGACAA	SI03053071	Hs_RAB8A_6		
AACAATCACGACAGCGTACTA	SI00116480	Hs_RAB8B_1	51762	RAB8B
CAGCACAATCTTAGACTCATA	SI02662261	Hs_RAB8B_6		
CAGCAGAGGTTAATATACTAA	SI02662814	Hs_RAB8B_7		
TTGCACGAGATATAATGACAA	SI03023972	Hs_RAB8B_8		
TAGATTGTTGATGCATTCTAA	SI00061747	Hs_RAB9A_3	9367	RAB9A
CACAGTCAATCTTCACCGAAA	SI00061754	Hs_RAB9A_4		
CAGCAGTGTATCATCTACTAA	SI02663234	Hs_RAB9A_5		
TTACTCAATAATGGCACCTTA	SI03021277	Hs_RAB9A_6		
CAGTCAGTAGTGTAAGAATAA	SI00115745	Hs_RAB9B_1	51209	RAB9B
CTGGAGGTAGATGGACGCTTT	SI00115759	Hs_RAB9B_3		
CAGGGTCTTCGTGCTGTTAAA	SI00115766	Hs_RAB9B_4		
AAGTTCGCTTATGAACCGTTA	SI03037153	Hs_RAB9B_6		
CAAATTCGGTTTCATATTCTA	SI00113701	Hs_RAB10_2	10890	RAB10

bioRxiv preprint doi: https://doi.org/10.64898/2026.03.29.715103; this version posted March 31, 2026. The copyright holder for this preprint (which was not certified by peer review) is the author/funder, who has granted bioRxiv a license to display the preprint in perpetuity. It is made available under aCC-BY 4.0 International license.

AAGGGACAAACTAGTAGGTTT	SI00113708	Hs_RAB10_3		
AAGCACTGATGTAACTTGCTA	SI00113715	Hs_RAB10_4		
AAGTGTGATATGGACGACAAA	SI03037076	Hs_RAB10_6		
TCGAGCTATAACATCAGCATA	SI00066801	Hs_RAB11A_4	8766	RAB11A
AAGAGTAATCTCCTGTCTCGA	SI00301553	Hs_RAB11A_5		
CGAAATGAGTTTAATCTGGAA	SI02655247	Hs_RAB11A_6		
AAGAGCGATATCGAGCTATAA	SI02663206	Hs_RAB11A_7		
CACGGACGGACAGAAGCCCAA	SI00061145	Hs_RAB11B_1	9230	RAB11B
CGAGTTCAACCTGGAGAGCAA	SI00061166	Hs_RAB11B_4		
CCGCATCACCTCCGCGTACTA	SI02655296	Hs_RAB11B_5		
CCGCATCGTGTCACAGAAACA	SI02662695	Hs_RAB11B_6		
CACTAATGAAGTGCAAATAAA	SI00486192	Hs_LOC201475_1	201475	RAB12
ATGGGAGAAATGAAAGGTATA	SI00486199	Hs_LOC201475_2		
CAGCATTACCTCAGCTTATTA	SI00486206	Hs_LOC201475_3		
CACGCTGCTGTCCTAAACAAA	SI00486213	Hs_LOC201475_4		
TAGGTAGATGGTAAAGAGAAT	SI00044912	Hs_RAB13_3	5872	RAB13
CAGGGCAAACATAAATGTAAA	SI02662149	Hs_RAB13_7		
ATGGTCTTTCTTGGTATTAAA	SI02662702	Hs_RAB13_8		
CAAGAGCGGTTCAAGACAATA	SI03053813	Hs_RAB13_9		
CACGTTAATAGAGGTAGTACA	SI00115346	Hs_RAB14_1	51552	RAB14
TCCATTGATCCCGTATCTTAA	SI00115353	Hs_RAB14_2		
AACGGTTCACGTCTAGCCAAA	SI00115360	Hs_RAB14_3		
AGGAATCGAACTAACAATACA	SI00115367	Hs_RAB14_4		

bioRxiv preprint doi: https://doi.org/10.64898/2026.03.29.715103; this version posted March 31, 2026. The copyright holder for this preprint (which was not certified by peer review) is the author/funder, who has granted bioRxiv a license to display the preprint in perpetuity. It is made available under aCC-BY 4.0 International license.

AAGGACTATATTTGTACTGTA	SI00697235	Hs_RAB15_1	376267	RAB15
CTGCACCAACCTCAACATTAA	SI00697242	Hs_RAB15_2		
CAGGTGCTTCAGAGAAGGAAA	SI00697249	Hs_RAB15_3		
TAGGAAGAGGGTCATTCTTAA	SI00697256	Hs_RAB15_4		
TCGCCTGAGATATAAGTTGTA	SI00133756	Hs_RAB17_1	64284	RAB17
CAGCCTCTGGACAGAGAGGAA	SI00133770	Hs_RAB17_3		
AAGTGAGATCCTGGAAGTGAA	SI00133777	Hs_RAB17_4		
CCAGGTGTCGGAGGTGTTCAA	SI03075569	Hs_RAB17_5		
ACAGGGTGTTATATTAGTTTA	SI00130473	Hs_RAB18_1	22931	RAB18
ACCAACTTGTACAGACTAATA	SI02662156	Hs_RAB18_7		
CCAGGCCAATTTATAACTAAA	SI02662709	Hs_RAB18_8		
AGGCCTGAAATTTGCACGAAA	SI03045161	Hs_RAB18_9		
TCCCTTGATATTGACGGCAAA	SI00697263	Hs_RAB19B_1	401409	RAB19
TTGACTATTTGTTCAAGATTA	SI00697270	Hs_RAB19B_2		
CTGGACCAAGAGTTCCCATTA	SI00697277	Hs_RAB19B_3		
CACGATTGGAGTGGACTTTAC	SI00697284	Hs_RAB19B_4		
CAGGCAAGCTGTGATCTGAAA	SI00119700	Hs_RAB20_1	55647	RAB20
CGACGTGTTCATTTATACAAA	SI00119707	Hs_RAB20_2		
CAGTGGATATATCCAGTCATA	SI00119714	Hs_RAB20_3		
CAGCAGAATGTTGGAGTGGAA	SI00119721	Hs_RAB20_4		
ATGGCTCTAGTCAGCCGGGAA	SI00109032	Hs_RAB21_4	23011	RAB21
CAGGCCCGTAACTGTCTACTA	SI02662177	Hs_RAB21_5		
CGAGAACAAGTTTAACGACAA	SI02662730	Hs_RAB21_6		

bioRxiv preprint doi: https://doi.org/10.64898/2026.03.29.715103; this version posted March 31, 2026. The copyright holder for this preprint (which was not certified by peer review) is the author/funder, who has granted bioRxiv a license to display the preprint in perpetuity. It is made available under aCC-BY 4.0 International license.

CAAGGCGAGGTGTACAGATTA	SI03054548	Hs_RAB21_7		
TTGGATTATTAAGCTGACTAA	SI00127358	Hs_RAB22A_4	57403	RAB22A
CAGGTTTAATTTGATGGTCTA	SI02662184	Hs_RAB22A_5		
AAGGCCTATCAGCCAATTAAA	SI02662737	Hs_RAB22A_6		
TGCCTTAGCACCAATGTACTA	SI03118584	Hs_RAB22A_7		
ATGATAAACTCTAGACATATA	SI00114989	Hs_RAB23_1	51715	RAB23
AACTAACGCATTCAAGTAGTA	SI03030391	Hs_RAB23_7		
AGCAGCTGTAGCATACCCTAA	SI03042725	Hs_RAB23_8		
GAGCGACAAATTCAAGTTAAT	SI03103030	Hs_RAB23_9		
CCAGGTGATGACAGAGGACAA	SI00149723	Hs_RAB24_2	53917	RAB24
CAAGGTGATGTCGGTCGGAGA	SI03054751	Hs_RAB24_5		
CAGCAGCTTTGAGCGAGCAAA	SI03065713	Hs_RAB24_6		
GGCCATGAGTAGAATCTACTA	SI03105704	Hs_RAB24_7		
GAGCTCTATGACCATGCTGAA	SI00126084	Hs_RAB25_1	57111	RAB25
AGGGTTGAGGGCATTGAGCAA	SI00126091	Hs_RAB25_2		
ATGGAACTGAGGAAGATTATA	SI02644082	Hs_RAB25_3		
AAGGTGGTGCTGATCGGCGAA	SI03036544	Hs_RAB25_5		
CACGCTCCACCTTGCACTCAA	SI00105084	Hs_RAB26_4	25837	RAB26
TCCGGCTGCATGATTACGTTA	SI02663262	Hs_RAB26_5		
CCGCAGTGTTACCCATGCCTA	SI02663269	Hs_RAB26_6		
CAGCGCCAAGACGGGCCTCAA	SI03067029	Hs_RAB26_7		
CCCATTAGACCTACGAATAAA	SI00065534	Hs_RAB27A_3	5873	RAB27A
AGAGATCTTAATGGCTATATA	SI00065541	Hs_RAB27A_4		

bioRxiv preprint doi: https://doi.org/10.64898/2026.03.29.715103; this version posted March 31, 2026. The copyright holder for this preprint (which was not certified by peer review) is the author/funder, who has granted bioRxiv a license to display the preprint in perpetuity. It is made available under aCC-BY 4.0 International license.

AAGATAGATGTTCATATTGAA	SI02662744	Hs_RAB27A_7		
ATGCCTCTACGGATCAGTTAA	SI03050075	Hs_RAB27A_8		
ACCGAATGGATCTTCAGGGAA	SI00060424	Hs_RAB27B_2	5874	RAB27B
TAAGCTGTACTAGAATGAATA	SI02662751	Hs_RAB27B_6		
AAACGTGTGGTTTATAATGCA	SI03026205	Hs_RAB27B_7		
TAGGAATAGACTTTCGGGAAA	SI03111486	Hs_RAB27B_8		
ACCCTTCAAATTTGGGATATA	SI00061705	Hs_RAB28_1	9364	RAB28
TTGGAGCATATGCGAACAATA	SI03024686	Hs_RAB28_5		
ACAGGGAGTCCTCTTGGTATA	SI03037895	Hs_RAB28_6		
CCGGGAAGACCTCCTTAACTA	SI03082394	Hs_RAB28_7		
CTCTAGGATGGTGATTTATAA	SI00057911	Hs_RAB7L1_3	8934	RAB7L1
AAGAGCTTAATCCTCATGATA	SI00057918	Hs_RAB7L1_4		
TAGGCACTTAGTCATAGGAAA	SI02662247	Hs_RAB7L1_5		
AACTTCTAACGTCATAATTAA	SI02662807	Hs_RAB7L1_6		
TAGGGCTTCGGTGGTAATCAT	SI00106512	Hs_RAB30_2	27314	RAB30
CTGCGGGAGATAGAACAATAT	SI00106526	Hs_RAB30_4		
AAGAGAGATTTCGGTCCATTA	SI02662191	Hs_RAB30_7		
TCGGTCCATTACCCAGAGTTA	SI02662758	Hs_RAB30_8		
CGGGCACATTAGGCAGTTGAA	SI00092750	Hs_RAB31_3	11031	RAB31
CAGGATTCACATAAACATTGT	SI00092757	Hs_RAB31_4		
AAGGAATACGCTGAATCCATA	SI02638230	Hs_RAB31_5		
CAGCTGTTATCGTGTATGATA	SI03068520	Hs_RAB31_6		
TTGCCATCATATGGAAGATAA	SI00092232	Hs_RAB32_1	10981	RAB32

bioRxiv preprint doi: https://doi.org/10.64898/2026.03.29.715103; this version posted March 31, 2026. The copyright holder for this preprint (which was not certified by peer review) is the author/funder, who has granted bioRxiv a license to display the preprint in perpetuity. It is made available under aCC-BY 4.0 International license.

TGGGTTACAGATGTCATGTTA	SI00092246	Hs_RAB32_3		
CTCTGCAAAGGATAACATAAA	SI02662198	Hs_RAB32_7		
CACCAAAGCTTTCCTAATGAA	SI02662765	Hs_RAB32_8		
AAGCATGGTCGAGCATTACTA	SI00068516	Hs_RAB33A_1	9363	RAB33A
AAACGATATAAATACAAGATA	SI03026128	Hs_RAB33A_5		
CGGCGTGGACTTCAGGGAGAA	SI03086517	Hs_RAB33A_6		
TGCCGTGGTCTTCGTCTATGA	SI03118521	Hs_RAB33A_7		
AAGGCACAACTCTAATTGCTA	SI00140455	Hs_RAB33B_2	83452	RAB33B
GCCGAGTTGCTTTCTTACTAA	SI00140469	Hs_RAB33B_4		
CTCCCGCATCTTCAAGATAAT	SI00301574	Hs_RAB33B_5		
AATGACCATGTGGAAGCTATA	SI03037356	Hs_RAB33B_6		
CCGCGTAATCGTAGGAACTAT	SI00141428	Hs_RAB34_1	83871	RAB34
CCGGGTTATTGTAGGAACCAT	SI00141435	Hs_RAB34_2		
CCGGGCCGCTCTGTAATGGTA	SI00141442	Hs_RAB34_3		
ATGCATTGCATCAACCTACTA	SI00141449	Hs_RAB34_4		
AGGGTGTGCTTGCAAATTCAA	SI00092624	Hs_RAB35_2	11021	RAB35
CAGTTTCGTGCCGTTATTTAA	SI02662205	Hs_RAB35_5		
CACGATCGGAGTGGATTTCAA	SI03059917	Hs_RAB35_6		
CAGTGCCGAGTCCTTTGTCAA	SI03073105	Hs_RAB35_7		
GTGGCCATTCTTGTAGTTATA	SI00069965	Hs_RAB36_1	9609	RAB36
AGGCACTGTGGTGATCCCATA	SI02634058	Hs_RAB36_5		
CAGCTCAGCGATTGCCAAATA	SI02634065	Hs_RAB36_6		
CAGGGAGATGCAGGCCGAGTA	SI03071103	Hs_RAB36_7		

bioRxiv preprint doi: https://doi.org/10.64898/2026.03.29.715103; this version posted March 31, 2026. The copyright holder for this preprint (which was not certified by peer review) is the author/funder, who has granted bioRxiv a license to display the preprint in perpetuity. It is made available under aCC-BY 4.0 International license.

AAGGCGGATATGAGCAGCGAA	SI00159362	Hs_RAB37_2	326624	RAB37
CTGTATGACATCACCAACAAA	SI00159376	Hs_RAB37_4		
CCGAAGCGTCACCCATGCTTA	SI03079902	Hs_RAB37_5		
CCGAGACTATGTAGAGTCCCA	SI03080084	Hs_RAB37_6		
CTCCAAGTTAAGTCTCCCTAA	SI00133224	Hs_RAB38_3	23682	RAB38
ACGAGGGTCTATTACCGAGAA	SI00133231	Hs_RAB38_4		
ATGATTTGGACTCCAAGTTAA	SI02663255	Hs_RAB38_5		
TGGGATATCGCAGGTCAAGAA	SI03121013	Hs_RAB38_6		
TCAGTTCAGGATAAATACCAA	SI00118552	Hs_RAB39_3	54734	RAB39
AAGGTTACAAACCCACACCAA	SI00118559	Hs_RAB39_4		
CAGGAGCGGTTCAGATCAATA	SI02663276	Hs_RAB39_5		
TAGCTTCACAACGTCAAGTTA	SI02663283	Hs_RAB39_6		
TTGGATCAGTTCACAGCATTA	SI00157143	Hs_RAB39B_1	116442	RAB39B
CAGGAGGCACTGTAACCCTTA	SI00157150	Hs_RAB39B_2		
TTGGATTAGATTGTCTCATAT	SI00157157	Hs_RAB39B_3		
AAAGATTATGAGCTCTACTTA	SI02648898	Hs_RAB39B_5		
CAGAAGAGCAAAGAATAATAA	SI00149443	Hs_RAB40A_1	142684	RAB40A
TCGCATTGTAGATTCAAGAAA	SI00149450	Hs_RAB40A_2		
TAGGCTGAATAATCTCCTGTA	SI00149457	Hs_RAB40A_3		
ATGGGAGGAAGAAATAGTACA	SI00149464	Hs_RAB40A_4		
ACGGTGATATTGGAGTGATTA	SI00092162	Hs_RAB40B_3	10966	RAB40B
CCGTCGGACTTCGAATTTCTA	SI02662212	Hs_RAB40B_5		
TCGGCGAATGCTGCTTGCGAA	SI02662772	Hs_RAB40B_6		

bioRxiv preprint doi: https://doi.org/10.64898/2026.03.29.715103; this version posted March 31, 2026. The copyright holder for this preprint (which was not certified by peer review) is the author/funder, who has granted bioRxiv a license to display the preprint in perpetuity. It is made available under aCC-BY 4.0 International license.

CCCGGCGGGCATCGACTACAA	SI03078600	Hs_RAB40B_7		
TCCGGTGAAGAGCTACGACTA	SI00129941	Hs_RAB40C_2	57799	RAB40C
TCCGGGAATCTTGGTCGGAAA	SI02662219	Hs_RAB40C_5		
CAGTAACGGGATCGACTACAA	SI02662779	Hs_RAB40C_6		
CCGCGCGTACGCAGAGAAGAA	SI03081092	Hs_RAB40C_7		
AAGATTGATTTGGATAACAAA	SI00697431	Hs_RAB41_1	347517	RAB41
GTGGTTGTCTATGACATTACA	SI00697438	Hs_RAB41_2		
ACGGTTGAAATCGAACTGGAA	SI00697445	Hs_RAB41_3		
CAGGAGCGCTTTCACAGCCTA	SI00697452	Hs_RAB41_4		
AAGGGTTCATATAAACAGTAT	SI00642467	Hs_MGC45806_1	115273	RAB42
CAGGAAGTCCTTTGAACACAT	SI00642474	Hs_MGC45806_2		
CTGCTGGTCTTTGATGTGACA	SI00642481	Hs_MGC45806_3		
CCGGCTCATCCACAAGACCCA	SI00642488	Hs_MGC45806_4		
AAACCATTAAACAGATTTCTA	SI00572404	Hs_LOC440374_1	339122	RAB43
ACCTATTTATGAATATACAAA	SI00572411	Hs_LOC440374_2		
CAGAGGTATCACAGTGACAAA	SI00697487	Hs_RAB43_1		
CCGAGCGTGGGTCCCAGTCTA	SI00697494	Hs_RAB43_2		

bioRxiv preprint doi: https://doi.org/10.64898/2026.03.29.715103; this version posted March 31, 2026. The copyright holder for this preprint (which was not certified by peer review) is the author/funder, who has granted bioRxiv a license to display the preprint in perpetuity. It is made available under aCC-BY 4.0 International license.

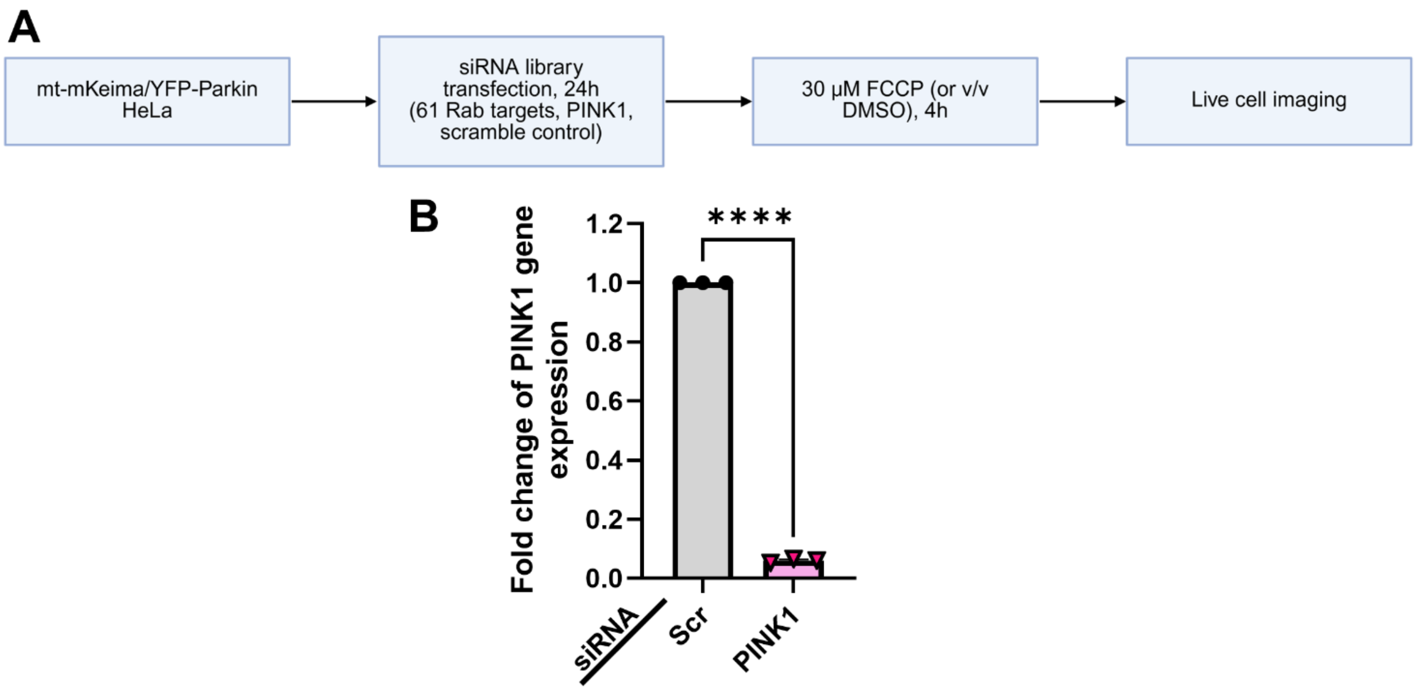

Supplementary Figure 2: Rab protein siRNA mitophagy screen protocol workflow and validation. (**A**) Outline of Rab family siRNA mitophagy screening protocol. HeLa cells stably expressing mt-mKeima and YFP-Parkin were plated onto 96-well plates pre-stamped with an siRNA library spanning the Rab family of proteins, along with a scramble negative control siRNA and a PINK1 siRNA as a positive control for mitophagy impairment. Following 24 h of transfection, cells were treated with FCCP (or an equivalent volume of DMSO vehicle control) for 4 h to induce mitophagy, followed by live-cell imaging for mitophagy analysis. Figure generated with BioRender. (**B**) RNA was collected for qRT-PCR analysis of PINK1 mRNA expression in mt-mKeima/YFP-parkin HeLa cells following reverse transfection with siRNA against PINK1 (siPINK1) or the scramble control (siScr) for 24 h. Internal control for normalization was GAPDH. ****$p < 0.0001$, as determined by unpaired t-test. $n = 3$ biological replicates. Data are presented as mean ± SEM.

bioRxiv preprint doi: https://doi.org/10.64898/2026.03.29.715103; this version posted March 31, 2026. The copyright holder for this preprint (which was not certified by peer review) is the author/funder, who has granted bioRxiv a license to display the preprint in perpetuity. It is made available under aCC-BY 4.0 International license.

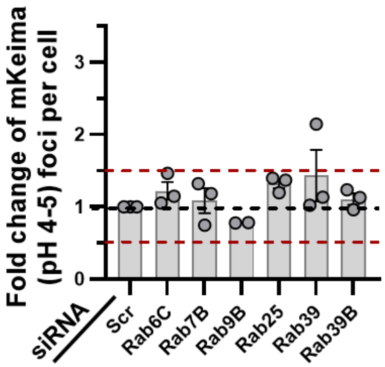

Supplementary Figure 3: siRNA against Rab proteins not expressed in HeLa cells did not alter mitophagy based on thresholding parameters selected for the siRNA screen. Endogenous expression levels of Rab family proteins were collected from the Human Protein Atlas. Candidates in the Rab family siRNA library utilized in mitophagy screening with no endogenous expression in HeLa cells were separated from candidates expressed in HeLa (Figure 2) and instead are displayed here. siRNAs targeting these non-expressed candidates had no impact on mitophagy based on the selected cutoff of a 50% increase or decrease in fold change of mt-mKeima (pH 4-5) foci per cell. n = 3 replicates (except Rab9B: n = 2 replicates).

bioRxiv preprint doi: https://doi.org/10.64898/2026.03.29.715103; this version posted March 31, 2026. The copyright holder for this preprint (which was not certified by peer review) is the author/funder, who has granted bioRxiv a license to display the preprint in perpetuity. It is made available under aCC-BY 4.0 International license.

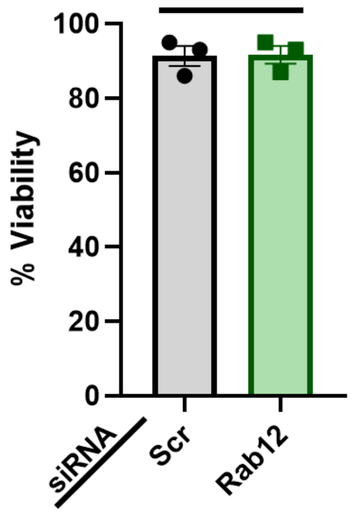

Supplementary Figure 4: Cell viability analysis in mt-mKeima/YFP-Parkin HeLa cells. The trypan blue exclusion assay was used to quantify percent viability in mt-mKeima/YFP-Parkin HeLa cultures transfected with scramble (Scr) siRNA or siRNA against Rab12 for 72 h. No difference in viability was observed with Rab12 knockdown relative to siScr transfection control. Data was analyzed using an unpaired t-test. $n = 3$ biological replicates. Data are presented as mean ± SEM.

bioRxiv preprint doi: https://doi.org/10.64898/2026.03.29.715103; this version posted March 31, 2026. The copyright holder for this preprint (which was not certified by peer review) is the author/funder, who has granted bioRxiv a license to display the preprint in perpetuity. It is made available under aCC-BY 4.0 International license.

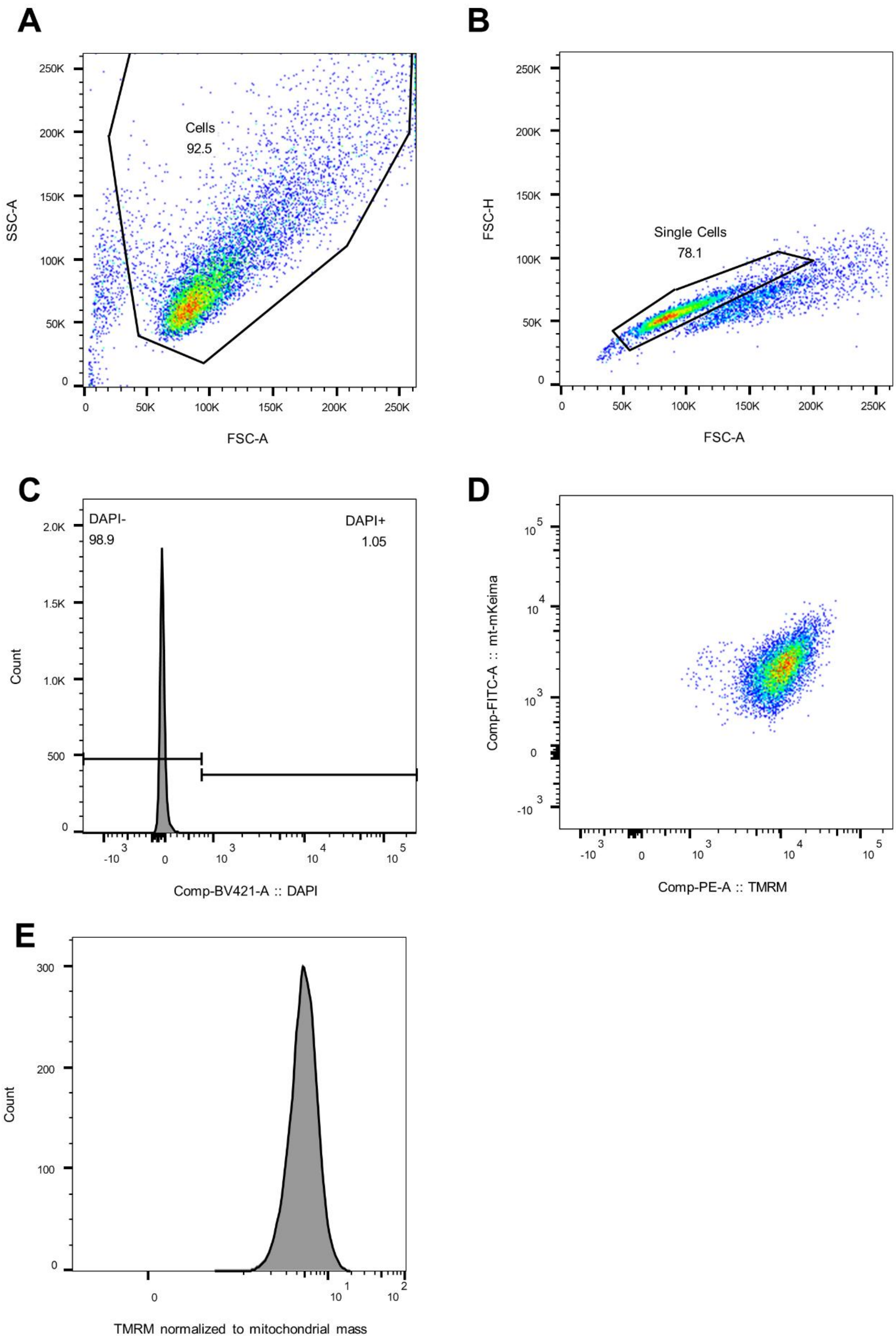

bioRxiv preprint doi: https://doi.org/10.64898/2026.03.29.715103; this version posted March 31, 2026. The copyright holder for this preprint (which was not certified by peer review) is the author/funder, who has granted bioRxiv a license to display the preprint in perpetuity. It is made available under aCC-BY 4.0 International license.

Supplementary Figure 5: Sample of siScr HeLa cells demonstrating gating strategy for flow cytometry after applying the compensation matrix. (A) FSC/SSC gated to exclude debris. (**B**) Single cells gated in plot of FSC-A/FSC-H, excluding doublets and aggregates. (**C**) Dead cells excluded by selecting events negative for DAPI. (**D**) Parameters of interest, TMRM signal versus mt-mKeima. (**E**) Histogram of the derived parameter - TMRM signal divided by mt-mKeima - representing TMRM normalized to mitochondrial mass per cell.

bioRxiv preprint doi: https://doi.org/10.64898/2026.03.29.715103; this version posted March 31, 2026. The copyright holder for this preprint (which was not certified by peer review) is the author/funder, who has granted bioRxiv a license to display the preprint in perpetuity. It is made available under aCC-BY 4.0 International license.

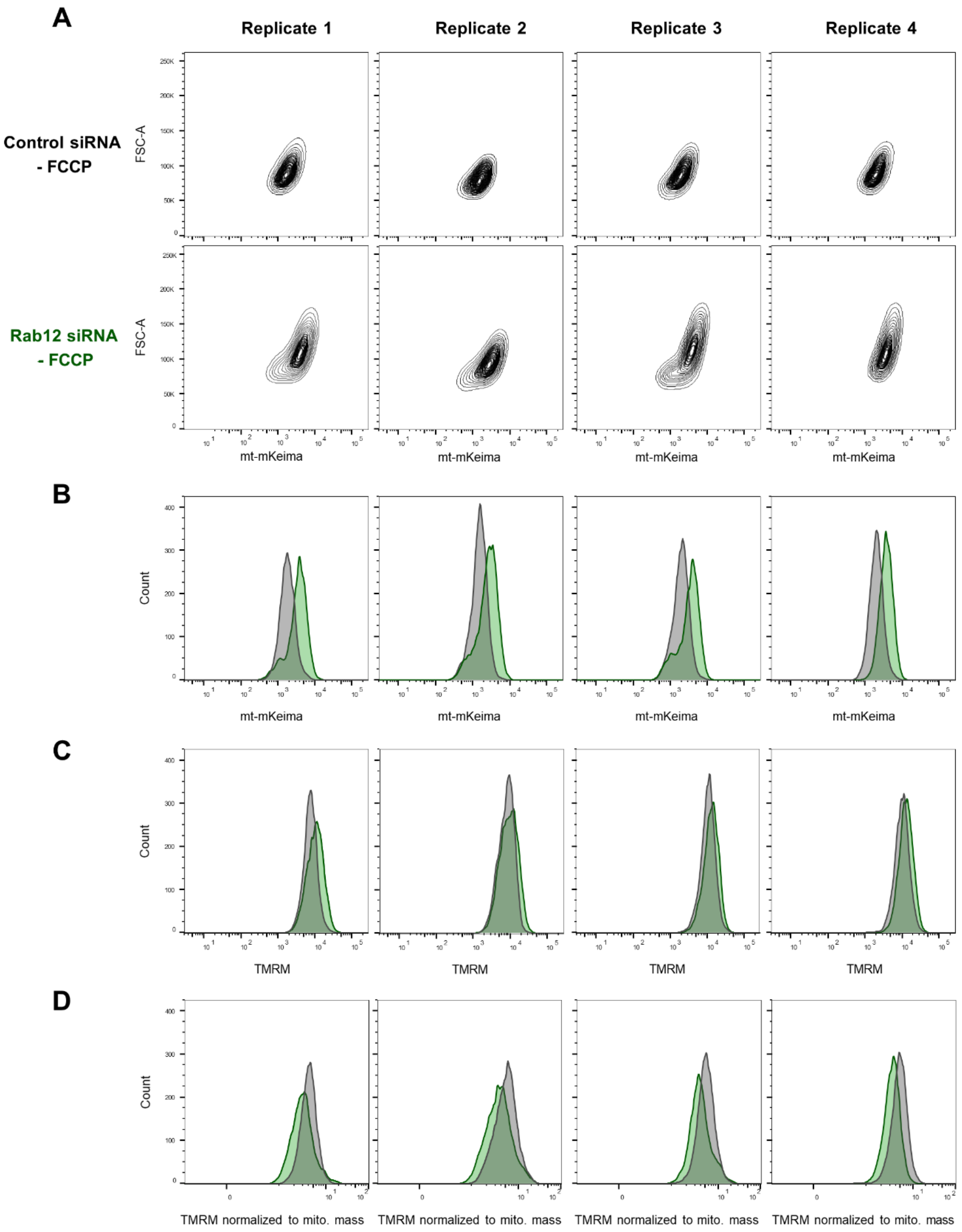

bioRxiv preprint doi: https://doi.org/10.64898/2026.03.29.715103; this version posted March 31, 2026. The copyright holder for this preprint (which was not certified by peer review) is the author/funder, who has granted bioRxiv a license to display the preprint in perpetuity. It is made available under aCC-BY 4.0 International license.

Supplementary Figure 6: Plots of siScr HeLa cells (gray) compared siRab12 HeLa cells (green) prior to FCCP exposure for four independent experiments. (**A**) mt-mKeima signal versus forward scatter (FSC). (**B**) Histograms of mt-mKeima signal. (**C**) Histograms of TMRM signal. (**D**) Histograms of the derived parameter, mt-mKeima divided by TMRM signal.

bioRxiv preprint doi: https://doi.org/10.64898/2026.03.29.715103; this version posted March 31, 2026. The copyright holder for this preprint (which was not certified by peer review) is the author/funder, who has granted bioRxiv a license to display the preprint in perpetuity. It is made available under aCC-BY 4.0 International license.

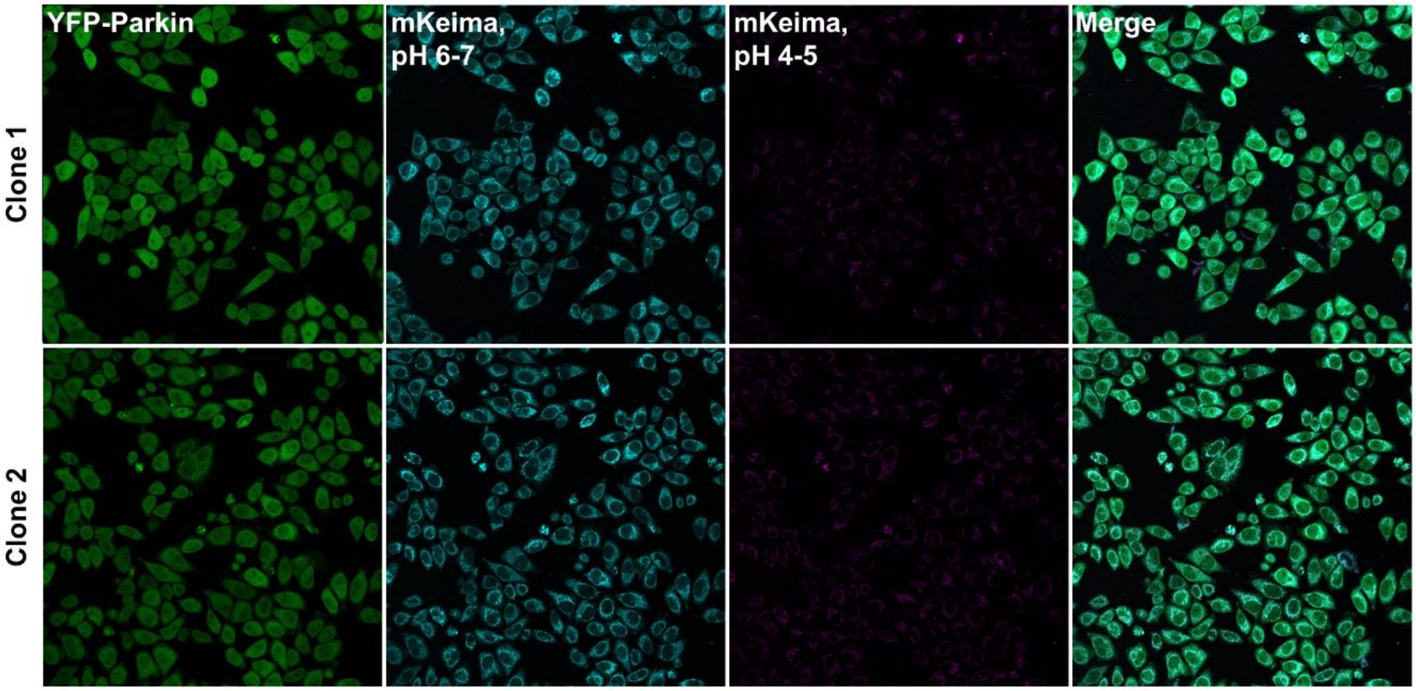

Supplementary Figure 7: Comparison of mt-mKeima/YFP-Parkin clones utilized in siRNA screening (Clone 1) and validation of Rab12's impact on mitochondrial homeostatic mechanisms (Clone 2). Cultures of mt-mKeima/YFP-Parkin HeLa cells exhibiting heterogeneous fluorescence levels of mt-mKeima and YFP-Parkin were single-cell sorted, and two separate clones were selected for siRNA screening (Clone 1) and validation of Rab12 impact on mitophagy (Clone 2). Representative 20X confocal images of the selected clones presented here display YFP-Parkin (green), mt-mKeima at neutral pH (cyan), and mt-mKeima at lysosomal pH (magenta), and were taken with identical laser settings to show similar fluorescence levels of both mt-mKeima and YFP-Parkin protein in each clone.
